# The Ascent of Alkyne Metathesis to Strategy-Level Status

**DOI:** 10.1021/jacs.1c08040

**Published:** 2021-09-14

**Authors:** Alois Fürstner

**Affiliations:** Max-Planck-Institut für Kohlenforschung, 45470 Mülheim/Ruhr, Germany

## Abstract

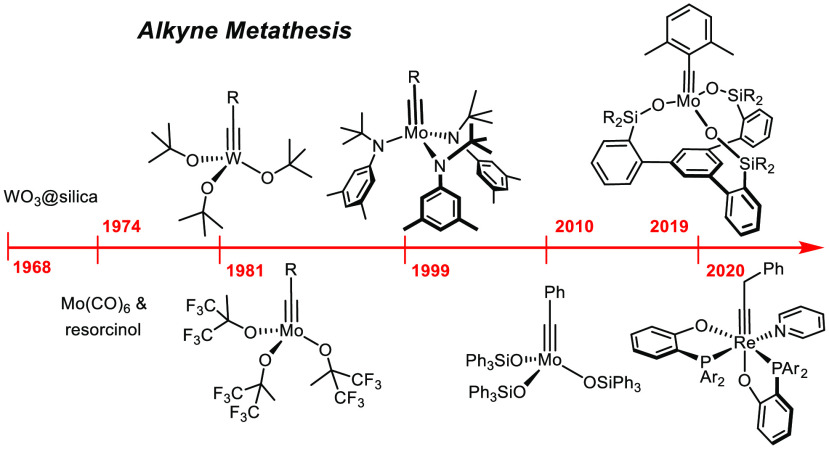

For numerous enabling features and strategic virtues, contemporary alkyne metathesis is increasingly recognized as a formidable synthetic tool. Central to this development was the remarkable evolution of the catalysts during the past decades. Molybdenum alkylidynes carrying (tripodal) silanolate ligands currently set the standards; their functional group compatibility is exceptional, even though they comprise an early transition metal in its highest oxidation state. Their performance is manifested in case studies in the realm of dynamic covalent chemistry, advanced applications to solid-phase synthesis, a revival of transannular reactions, and the assembly of complex target molecules at sites, which one may not intuitively trace back to an acetylenic ancestor. In parallel with these innovations in material science and organic synthesis, new insights into the mode of action of the most advanced catalysts were gained by computational means and the use of unconventional analytical tools such as ^95^Mo and ^183^W NMR spectroscopy. The remaining shortcomings, gaps, and desiderata in the field are also critically assessed.

## Introduction

Alkyne metathesis, that is, the redistribution of the alkylidyne units of a pair of acetylene derivatives with the aid of a transition-metal catalyst, has been known since the late 1960s.^1^ Although barely younger than olefin metathesis, it took a long time for this transformation to step out of the shadow of its exceptionally impactful sibling in order to gain a sharp profile in its own right.

As the early phase of alkyne metathesis has been thoroughly reviewed, it may suffice here to recapitalize only the major lines of development.^2−9^ The rather long initial “latency” period certainly has to do with the fact that the lead findings were somewhat fuzzy and incongruent.

The reaction was discovered with a heterogeneous catalyst (WO_3_/silica) that required very high temperatures (200–450 °C) to be operative.^1^ As such conditions hardly apply to any elaborate substrate, this system had little impact and truly heterogeneous catalysts in general remained largely unexplored.^2−9^

The first alkyne metathesis reaction in homogeneous phase used Mo(CO)_6_ and a phenol additive.^10^ This recipe was well received for its preparative convenience and continues to find occasional applications.^11^ Once again, however, fairly high temperatures are mandatory (160 °C in the original report), at which excess phenol is not necessarily an innocent bystander. It is hence hardly surprising that this method applies to robust substrates only and its functional group compatibility is inherently limited. Moreover the active species generated in situ defied direct inspection; therefore, this catalyst system did not lend itself to rational (rather than empirical) optimization.

**Scheme 1:**
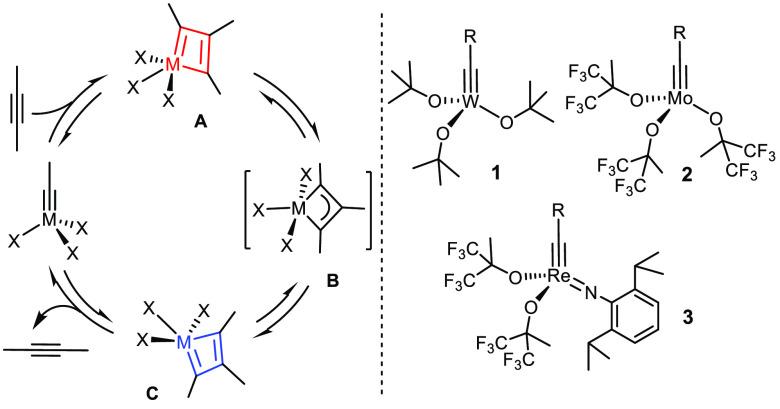
General Reaction Mechanism (Degenerate Setting) and Primordial Catalysts

This stunning lack of information on a reasonably popular catalyst system stood in stark contrast to the very detailed understanding of the organometallic principles. The advent of high-valent transition-metal alkylidyne complexes (“Schrock alkylidynes”) laid a solid ground for thorough mechanistic investigations.^2,12,13^ These studies provided compelling evidence for a sequence of [2 + 2] cycloaddition/cycloreversion steps being accountable for the reaction, as had been hypothesized before (Scheme 1).^14^ More precisely, the square-pyramidal metallacyclobutadiene tautomer **A** primarily formed converts into tautomer **C**, from which the product is released by [2 + 2] cycloreversion; this reorganization passes through a trigonal-bipyramidal intermediate (or transition state) **B**.^15^ Moreover, it became very soon very clear that the proper choice of ancillary ligands about the active alkylidyne unit is a critical determinant of catalytic activity.^2,12,13^

The tungsten alkylidyne complex **1a** (R = *t*Bu) (and its close relatives) developed by Schrock and co-workers was the historically first well-defined alkyne metathesis catalyst.^16−19^ The same group also showed that certain molybdenum alkylidynes are competent, provided they carry poorer π-donor ligands such as (per)fluorinated *tert*-alkoxides;^18,20,21^ as the original synthesis of complexes of type **2** was much less efficient than of their tungsten counterparts, this class of catalysts largely fell into oblivion and was rediscovered only much later (see below). Finally, rhenium alkylidynes such as **3** were found to be (moderately) active.^22−24^

The catalysts have undergone massive evolution since then; yet, all relevant alkyne metathesis catalysts used to date continue to be tungsten, molybdenum or rhenium alkylidynes; they are hence variations on the themes originally invented by Schrock in the (early) 1980s.

## Why Bother?

For the tremendous success of contemporary olefin metathesis,^25^ one might argue that there is little need for alkyne metathesis in general, not least because most (internal) alkynes are chemically more “expensive” than analogous olefins. Considering the certainly unequal substrate basis only, however, may lead to a distorted picture.

**Scheme 2:**
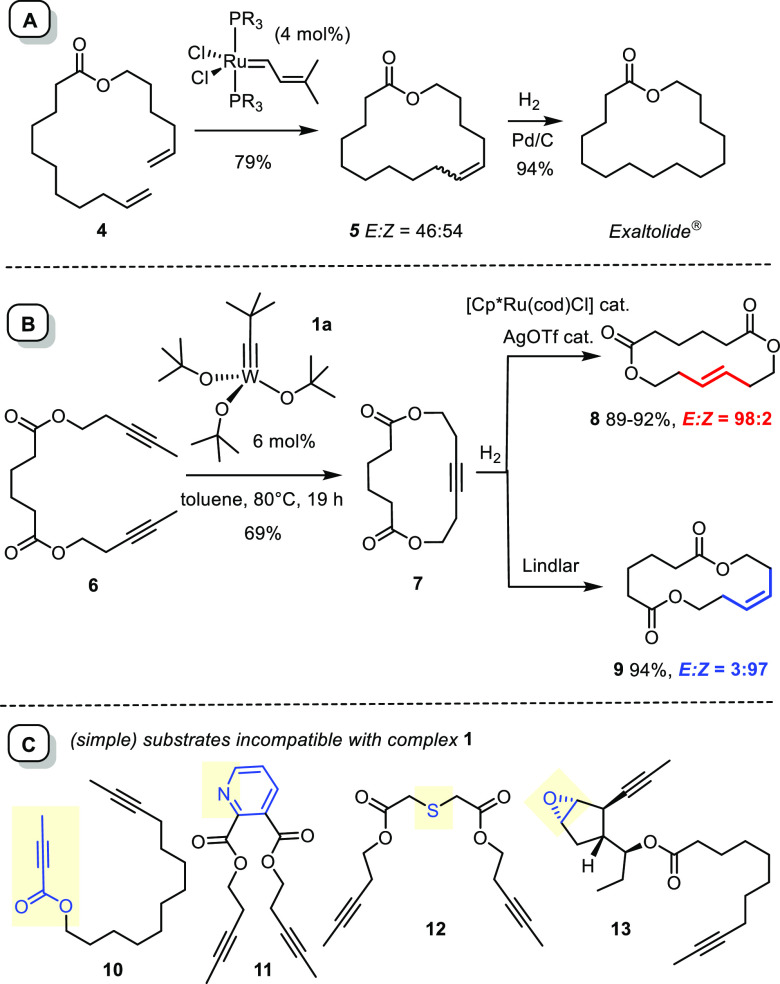
Lead Findings in Macrocycle Synthesis via RCM (A) or RCAM (B). Model Substrates Incompatible with the Tungsten Alkylidyne **1** (C)

Notwithstanding some skepticism in the early literature whether or not olefin metathesis applies to the formation of macrocyclic rings,^26^ our group was able to demonstrate that even conformationally unbiased dienes such as **4** cyclize well when treated with the then brand-new (and at the time not yet commercial) first-generation Grubbs catalyst;^27^ subsequent hydrogenation of product **5** furnished the musk odorant exaltolide in excellent overall yield (Scheme 2).^28,29^ While this discovery went a long way since then in our laboratory and elsewhere,^25,30−34^ this lead discovery actually revealed an important limitation too: cycloalkene **5** was obtained as an almost 1:1 mixture of the two alkene isomers, likely because the reversibility of RCM entails thermodynamic control. Though inconsequential in this particular case, it was immediately clear that applications of RCM-based macrocyclizations to more elaborate substrates will eventually face a serious handicap: substantial or even complete loss of precious material is possible as long as no control over the configuration of the newly formed double bond can be exerted.

The development of *kinetically Z*- or *E*-selective catalysts is arguably the best way forward. While much progress has been made in this direction,^35−37^ there is still considerable room for improvement; most notably, kinetic *E*-selectivity continues to lack a reasonably general solution.^38,39^ For this reason, we started to contemplate whether alkyne metathesis followed by stereoselective semireduction could be an alternative answer or not.

Proof-of-concept for the formation of macrocyclic rings by ring closing alkyne metathesis (RCAM) was attained with diyne **6** (Scheme 2).^40,41^ This particular substrate was transformed with the aid of **1a** into cycloalkyne **7**, which could then be converted into *Z*-alkene **9** or the corresponding *E*-alkene **8**(42,43) in high yield and excellent selectivity.

The ability to set the desired alkene geometry with high fidelity even at a late stage of a synthesis endeavor is arguably an asset of potentially strategic relevance.^44^ What is more, a triple bond offers innumerous possibilities for postmetathetic transformations other than semireduction. It was therefore reasonable for us to believe at the outset of our project that alkyne metathesis could serve as a springboard that allows a plethora of structural motifs to be attained. The reaction should certainly be considered as a serious alternative to stereoselective olefin metathesis; at the same time, however, it might become relevant far beyond this frontier as a valuable synthetic tool in its own right.

For this vista to become true, however, a number of massive challenges had to be met. As the formation of **7** had shown, the activity of complex **1** was rather modest; warming was necessary to ensure reasonable rates and the yield of product was not overly high either.^40^ A much more serious limitation was the narrow functional group tolerance of **1** and analogues.^41,45^ Although a few applications to target-oriented synthesis proved successful,^6,7,9,46−50^ substrates as simple as **10**–**13** were inadequate likely because the donor sites quench the activity of the Lewis-acidic tungsten catalyst and/or get activated and destroyed upon coordination to **1** (Scheme 2); electron-rich or -deficient substrates were not compliant either.^41^ An attempt to make the anticancer agent epothilone C by RCAM of diyne **18** followed by Lindlar reduction was therefore inconceivable with the toolbox available before the turn of the millennium (see below).

## A Knight’s Move

A much improved compatibility of the catalysts with functional groups of all sorts hence constituted the single most important goal to be attained at the outset of our venture. To this end, we decided early on to defer work on tungsten alkylidynes and redirect our efforts toward molybdenum-based systems for the lower intrinsic Lewis acidity of this transition metal.

**Scheme 3:**
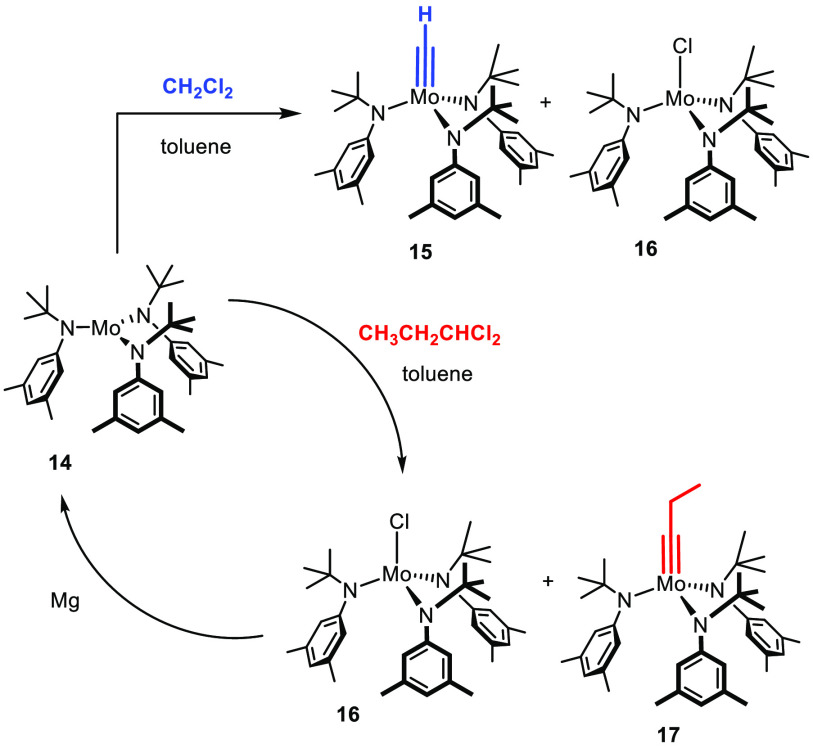
Molybdenum Alkylidynes Derived from a Mo(+3) Precursor: Basic and Advanced Format

**Scheme 4:**
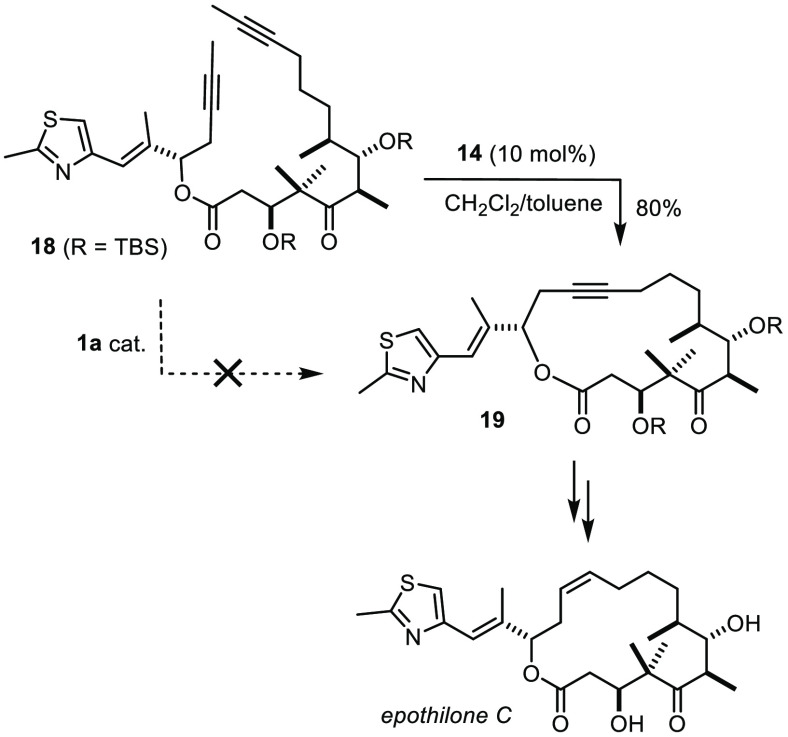
Total Synthesis of Epothilone as an Early Illustration of the Superior Tolerance of [Mo]-Based Catalysts

A first step was taken when we found that treatment of complex **14**(51) with CH_2_Cl_2_ generates an active species, which indeed shows a much improved tolerance, notably toward common heteroatom donor sites (Scheme 3).^52−54^ This aspect was illustrated by the successful application of **14**/CH_2_Cl_2_ to the formation of epothilone C which the tungsten alkylidyne **1a** was unable to reach (Scheme 4);^53,55^ additional case studies followed shortly thereafter.^6^ Even today, **14**/CH_2_Cl_2_ remains an indispensable tool, especially in cases in which encumbered alkynes need to be activated.^56−58^

On the basis of mechanistic studies which had shown that the C atom of CH_2_Cl_2_ gets transformed into the methylidyne unit of **15** on reaction with **14**,^53^ Moore and co-workers developed an important modification.^59,60^ By using higher *gem*-dihalides as the activating agents, they managed to obtain and isolate the corresponding alkylidyne complexes such as **17**; in combination with a reductive recycling strategy, this approach is also “economical” with regard to the molybdenum source. If one desires so, protonolysis of the fairly basic amide ligands in **17** with a phenol of choice (or, later, with an appropriate silanol) allows the ligand sphere to be adjusted and the catalytic properties to be fine-tuned. This system found numerous applications in polymer chemistry and material science.^5,61^

The chemical virtues notwithstanding, all catalysts derived from **14** come at a high “price”: this complex is extremely sensitive and must be prepared and handled with rigorous Schlenk techniques; moreover, it mandates an Ar atmosphere because it is even capable of activating N_2_ under mild conditions.^51^ The procedures for its preparation have to be strictly followed since the (redox) chemistry of low-valent molybdenum is intricate and by no means fully understood.^62^ Likewise, the solvents must be scrupulously dried; the presence of protic sites in the substrate to be metathesized is inconceivable. The step forward taken with **14**/CH_2_Cl_2_ or complexes such as **17** in terms of functional group compatibility hence came along with a step sideward (backward) with regard to handling.

## Silanolate Molybdenum Alkylidynes

Although the practicality of **14**/CH_2_Cl_2_ is poor, this system provided compelling evidence that alkyne metathesis reactions can be performed in complex settings without interference of common polar and apolar substituents. However, it was also clear that better access to the relevant catalysts had to be found without need to resort to **14** as the molybdenum source.

**Figure 1:**
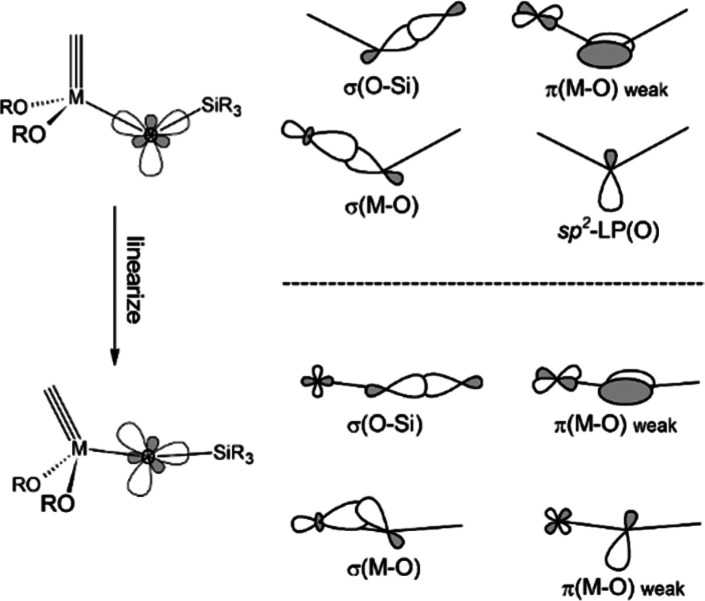
Angle-dependent metal–ligand bonding.

Apprehensive of the fact that the very first alkyne metathesis had used a catalyst adsorbed on silica,^1^ we conjectured that silanolates might be suitable ancillary ligands.^63−66^ They are poorer π-donors than (tertiary) alkoxides;^67^ moreover, the Si–O–Mo linkage is floppy and hence easy to stretch and bend (Figure 1): in so doing, the oxygen atom formally shuttles between the extremes of sp and sp^3^ hybridization, which gently tunes its donor ability.^68^

The adaptive electronic character of a silanolate ligand is arguably ideal in the context of alkyne metathesis.^63,64,69^ Note that the catalytic cycle shown in Scheme 1 consists of a sequence of elementary steps with *alternating electronic optima*: substrate binding and metallacycle formation are favored by a more Lewis-acidic central atom, whereas the retro-[2 + 2] step and product release are easier at a more electron-rich site. To ensure efficient turnover, these opposing needs must be properly balanced; the adaptiveness of silanolates provides an *intrinsic* handle to do so.^63,64^ At the same time, the Lewis acidity of the molybdenum alkylidyne unit itself will likely be tempered such that an attractive overall application profile might ensue. The fact that various (alkyne) metathesis catalysts had been successfully immobilized on silica surfaces or attached to polyoligomeric silsesquioxanes was also deemed encouraging:^70,71^ it is the silyloxy group of the first coordination sphere that basically determines the electronic structure of the metal center.^72^ In any case, the reasoning that silanolates might synergize with the operative molybdenum alkylidyne fragment largely proved correct: in terms of functional group compatibility, silanolate-bearing catalysts brought alkyne metathesis to a previously unknown level and continue to set the standards in the field.^6,63−65^

**Scheme 5:**
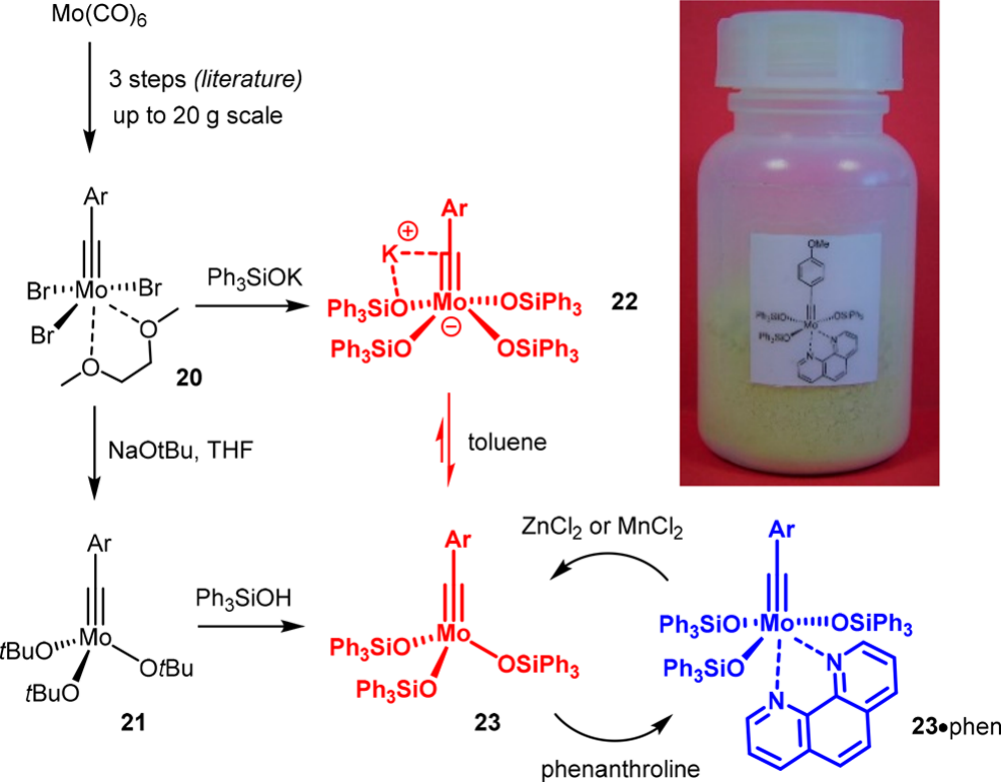
Improved Synthesis of Molybdenum Alkylidynes Exemplified by the Parent Silanolate Catalysts and the Bench-Stable Phenanthroline Adduct

In parallel, more proficient entries into molybdenum alkyidyne complexes were established. The currently best way adopts a route originally developed by Mayr and co-workers in which readily accessible Fischer-carbyne complexes are oxidized with Br_2_ to furnish Schrock tribromoalkylidynes such as **20** (Scheme 5).^73,74^ Although this step needs careful temperature control, it can be performed on a multigram scale. Compound **20** itself is catalytically inactive, as is the derived alkoxide complex **21**. Treatment of **20** with triphenylsilanolate affords the ate complex **22** (unless the addition is carefully controlled); because the uptake of the fourth ligand is reversible, **22** serves as a reservoir for the neutral alkylidyne **23** as the actual catalyst.^63,64^ Ate-complex formation is therefore no impediment; in certain cases, the slow release of the active species in solution is even beneficial (see below). If one so desires, however, this issue can be avoided by resorting to **21** as the substrate: treatment with the silanol of choice results in quantitative ligand exchange; no salt is formed and the liberated *tert*-butanol can simply be removed in vacuo. This procedure is presently our preferred route to the parent complexes **23** as well as to the more modern “canopy catalysts” discussed below.^75^ In this context, it is also important to mention that the renaissance of molybdenum alkylidynes endowed with partly fluorinated or perfluorinated alkoxide ligands, which the Schrock group had already pioneered but which had then found little echo, was also brought about by the improved access route passing through tribromides such as **20**.^8,76^

**Figure 2:**
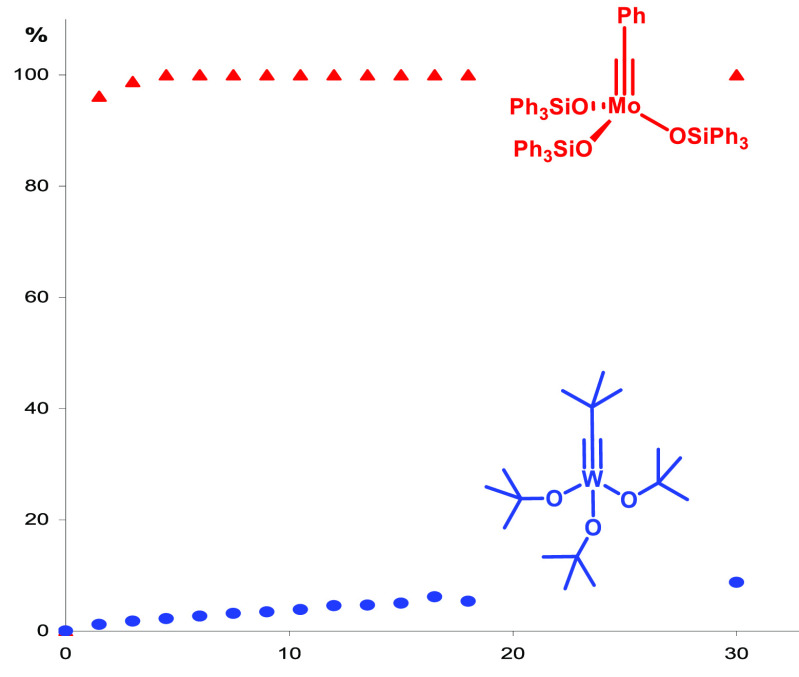
Benchmarking of the activity of complexes **23a** and **1a** (1 mol % each): formation PhC≡CPh from PhC≡CMe in the presence of MS 5 Å as 2-butyne scavenger in toluene at ambient temperature.

Figure 2 illustrates the high activity of these new catalysts by comparison with the Schrock alkylidyne **1a** as the classical reference point.^63,64^ The reasons for this improved performance are fairly well understood: it has recently been possible, for example, to isolate the metallacyclobutadiene **24** derived from **23** on reaction with excess 3-hexyne.^77^

**Figure 3:**
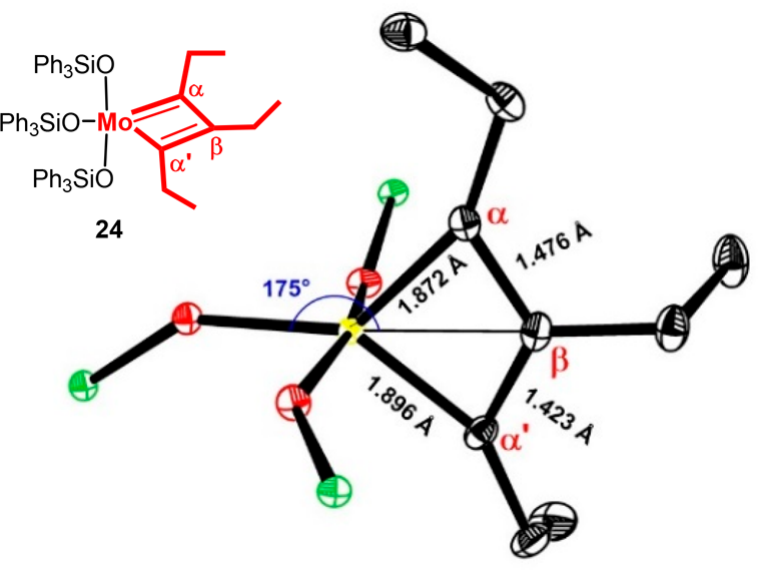
Core region of metallacyclobutadiene **24** in the solid state; Mo = yellow, O = red, Si = green.

The structure of this very sensitive complex in the solid state is highly instructive (Figure 3): it adopts a geometry in between square-pyramidal and trigonal-bipyramidal; although the bond lengths are uneven, the differences are small. In solution, the interconversion of this tautomer with the second tautomer necessary for productive cycloreversion (corresponding to **A** → **C** in Scheme 1) could not be frozen out even at −90 °C.^77^ It obviously takes very little for **24** to pass through the trigonal-bipyramidal rendition (**B**) at which the intermediates responsible for productive turn over converge. These spectroscopic and crystallographic data hence nicely explain the excellent reactivity of complex **23** and congeners.

For a reaction that is inherently reversible, it is necessary to perturb the equilibrium in order to reach quantitative conversion. We showed that addition of molecular sieves is a convenient way to do so, as they are capable of trapping 2-butyne (released when working with methyl-capped alkynes as the most common substrates) and hence allow the reaction to proceed to completion even at ambient temperature.^63,64^ This practice has been widely embraced.^6−9,78^

**Figure 4:**
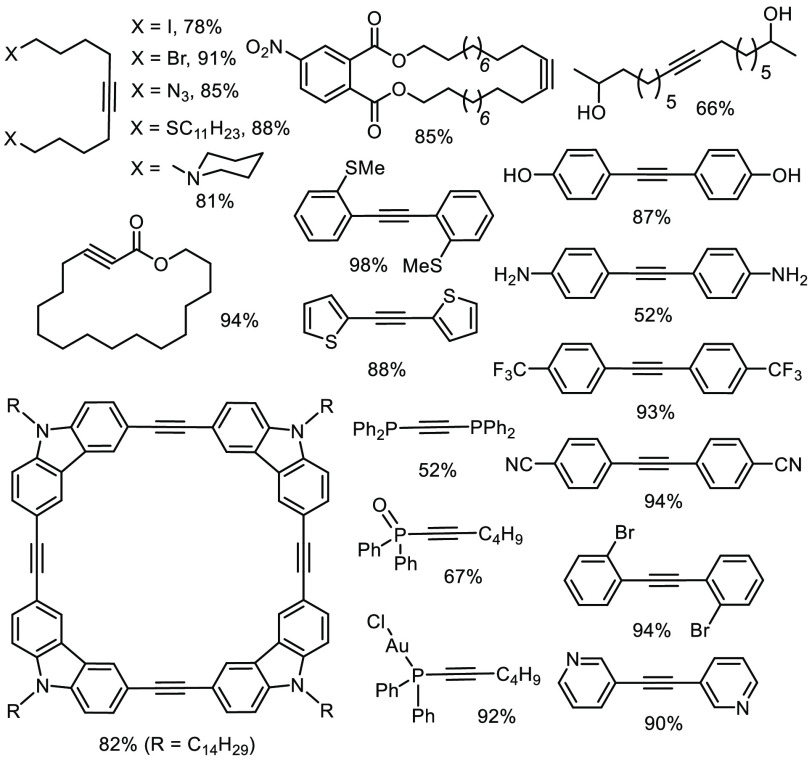
Survey of compatible functional groups

Figure 4 gives a noncomprehensive overview over functional groups compatible with **23** and relatives.^63−65^ A few comments may help to calibrate these results: while the tungsten complex **1** had failed with substrates carrying even moderately basic N atoms, S-donor sites, or common heterocyclic rings (Scheme 2), **23** is largely undisturbed by their presence. Likewise, **1** is incapable of metathesizing electron-rich (e.g., alkynylsilanes, -phosphines) or electron-deficient substrates (e.g., alkynoates),^41,79^ whereas **23** does so; this latter aspect proved enabling as witnessed by several total synthesis projects.^65,80−82^ At the meta level, this catalyst can even be compared to the famous ruthenium carbene complexes for olefin metathesis, the exquisite tolerance of which is highly appreciated;^25^ however, Grubbs-type catalysts often fail if the substrates contain amines, sulfides, phosphines. or nitriles. Moreover, phosphine-bearing ruthenium carbenes endanger azides, alkyl halides, epoxides, and the like.^25^ When seen against this backdrop, the compatibility of **23** comprising a non-noble metal atom in its highest oxidation state with all of these functionalities is deemed remarkable. On the other hand, Grubbs-type catalysts truly excel in the presence of protic groups and remain operative even in aqueous media.^25^ Although **23** does tolerate certain protic sites, especially when sterically hindered (see below), the sensitivity toward (moderately) acidic functionality in general remains the most eminent Achilles heel.

**Figure 5:**
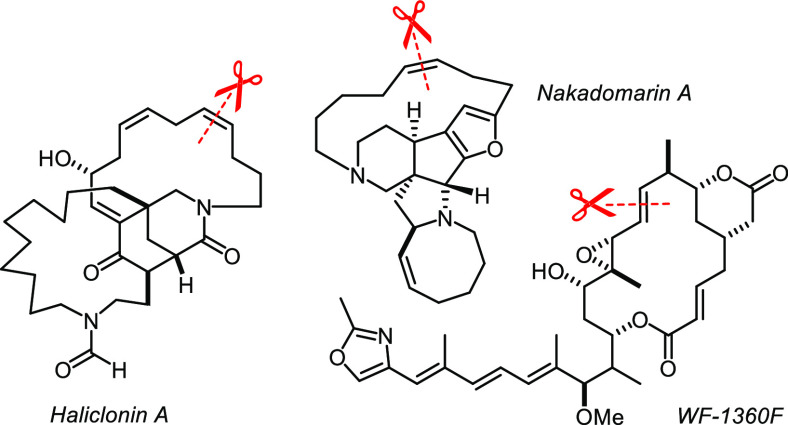
Selected applications of the bench-stable phenanthroline adduct **23**·phen in target-oriented synthesis.

**Figure 6:**
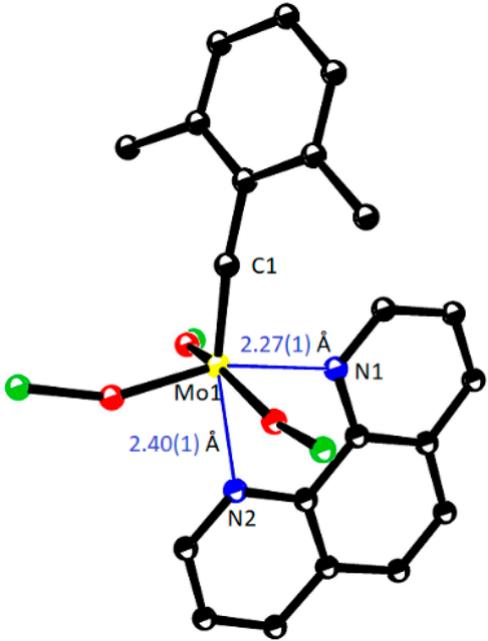
Core region of complex **23**·phen (Ar = 2,6-Me_2_C_6_H_3_): the uneven Mo–N bonds, reflecting the suboptimal orbital overlap between Mo1 and N2 and the *trans*-effect of the alkylidyne, render binding of the chelate ligand reversible.

Complex **23** and its relatives have an additional bonus in that they form bench-stable adducts with 1,10-phenanthroline or 2,2′-bipyridine (Scheme 5).^63,64^ Because the *trans*-effect of the alkylidyne weakens the opposing N···Mo bond in **23**·phen and the orbital overlap between Mo1 and N2 is not perfect on geometric grounds either (Figure 6), the stabilizing chelate ligand can be pulled off with the aid of ZnCl_2_ or MnCl_2_, and the active catalyst be released in solution.^63,64^ To be able to fill a highly active and superbly selective catalyst into a bottle (Scheme 5)—though in masked form—seemed elusive at the outset of our project. For the first time, adducts such as **23**·phen enable those practitioners who are less experienced with and/or equipped for the handling of highly sensitive complexes to leverage the power of contemporary alkyne metathesis catalysts. Recent total syntheses of haliclonin A,^83,84^ nakadomarin A,^85^ crysophaentin F,^86^ the tubulin inhibitor WF-1360F^87^ and a study toward crassin acetate^88^ illustrate the utility of these stabilized catalysts (Figure 5).

## A Functional Group Paradox

**Scheme 6:**
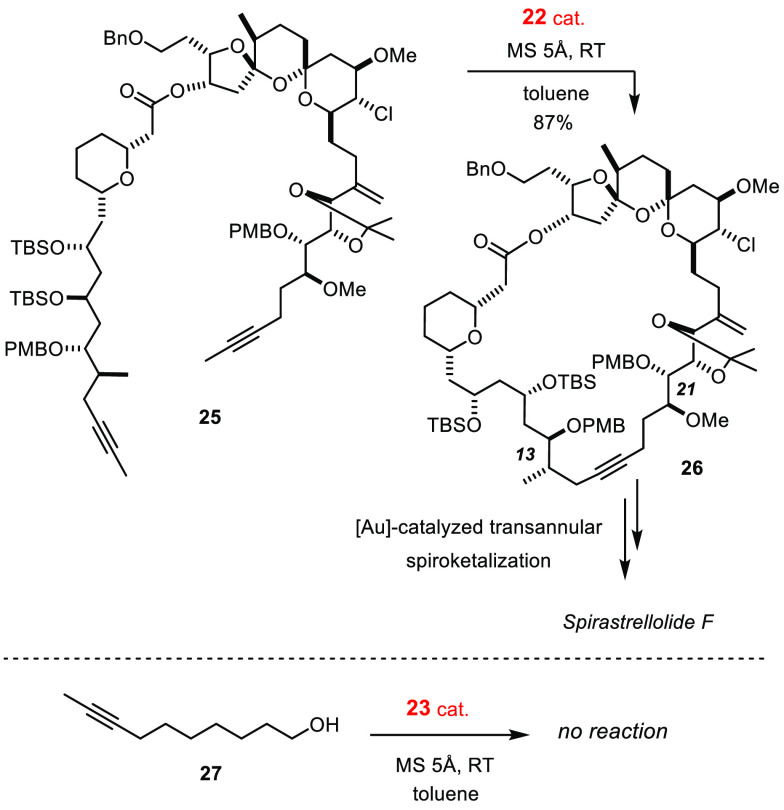
Functional Group “Paradox”

The comparison shown in Scheme 6 sheds light on a certain paradox: thus, the cyclization of the densely decorated diyne **25** with the aid of ate complex **22** (slowly releasing complex **23** in solution) to give **26** was fast (<30 min) and high yielding.^89^ Deprotection of the two −OPMB groups at C13 and C21 followed by spirocyclization upon activation of the triple bond with a gold catalyst paved the way to the intricate protein phosphatase inhibitor spirastrellolide F.^89^

In striking contrast, a substrate as simple as **27** carrying nothing but an unhindered primary −OH group proved unmanageable: if the alcohol replaces the silanolate ligands in **23**, the catalyst gradually or completely loses activity.^90,91^

## The “Canopy” Series

We conjectured that recourse to the chelate effect might help remedy this issue, at least in part, since simultaneous cleavage of three silanolate linkages is statistically less likely and partial solvolysis potentially reversible. A first foray, which used the readily available trisilanoles **28** and **29**, was partly unsuccessful yet encouraging.^92^ Partial cross-linking occurs on reaction of these conformationally flexible ligands with complex **17**: the resulting mixture of dimeric/oligomeric species, however, exhibits excellent catalytic properties. As expected, an improved—though certainly not perfect—compatibility with free −OH groups and other protic sites was noticed.^92^

**Scheme 7:**
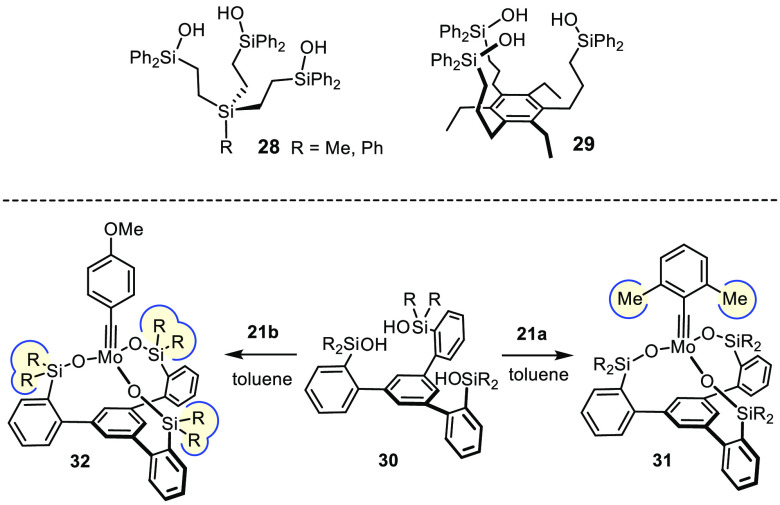
Evolution of the Tripodal Silanolate Ligand Framework: The “Canopy” Series”

Hence, the ligand design was revisited with the hope of forming catalysts that retain these virtues yet are structurally well-defined. The platform shown in Scheme 7 proved adequate: trisilanols **30** are sufficiently preorganized to ligate a single metal center but flexible enough to support the different intermediates passed through during a catalytic turnover.^75,77,93^ The preparation of ligands of this type is straightforward, scalable, and modular; the same design was independently pursued by the group of Lee.^94,95^

**Scheme 8:**
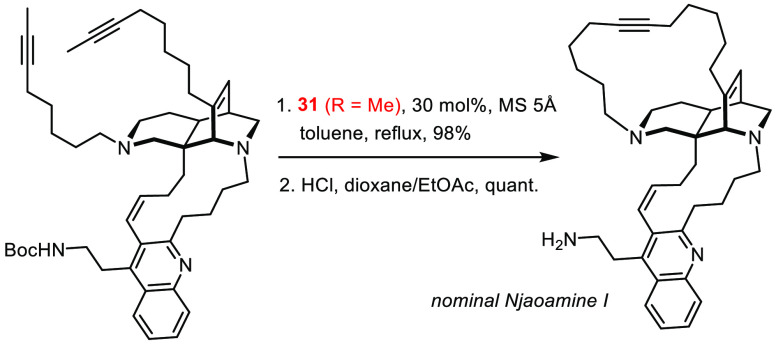


The derived “canopy” complexes of type **31** or **32** are indeed privileged catalysts. They combine the advantages of the parent silanolate-supported molybdenum alkylidynes **23** with a higher robustness against protic substituents including unprotected alcohols (for specific examples, see Schemes 16 and 17).^75^ Even a certain stability toward moisture was noticed; although there remains much room for improvement, the ability to perform alkyne metathesis reactions in technical grade solvents is deemed an important step toward a truly practical methodology.^75^ Their compatibility with numerous Lewis-basic groups is equally astounding if one considers that the operative metal alkylidyne comprises an early transition metal in its highest oxidation state: the finish of a total synthesis of nominal njaoamine I proves that complex **31** (R = Me) remains fully operative even in the presence of two different tertiary amines and a quinoline (Scheme 8).^96^ To properly assess this result, it may suffice to say that a single tertiary amine sufficed to quench the activity of first and second generation Grubbs-type catalysts in a very closely related RCM-setting en route to the sibling alkaloid ingenamine.^96^

For this promising application profile, the canopy catalysts have already been subject to intense experimental and computational scrutiny.^69,75,77,95^ Since a comprehensive discussion is beyond the scope of this Perspective, a portrait in “al fresco” style must suffice. Thus, the geometric constraints of the podand ligand framework lead to a slightly more Lewis-acidic Mo center, which favors substrate binding. This step is also strongly affected by the size of the substituents on the Si atoms forming a fence about the alkylidyne unit; small unbranched alkyl groups therefore provide a kinetic advantage. Complex **31** (R = Me) carrying lateral methyl substituents is currently the most active member of this series,^75^ although the homologues with higher unbranched alkyl groups retain appreciable reactivity.^97^ The missing steric protection, however, renders complex **31** susceptible to degradation by bimolecular coupling,^97^ which may explain why fairly high loadings had to be used in some demanding applications.

## An Unorthodox Mechanism

The arguably most striking aspect, however, concerns the reaction mechanism itself. The corset of the ligand framework prevents the isomerization of the metallacyclobutadiene **D** primarily formed into a second tautomer from occurring; therefore, the canonical course of alkyne metathesis as shown in Scheme 1 is blocked.

**Scheme 9:**
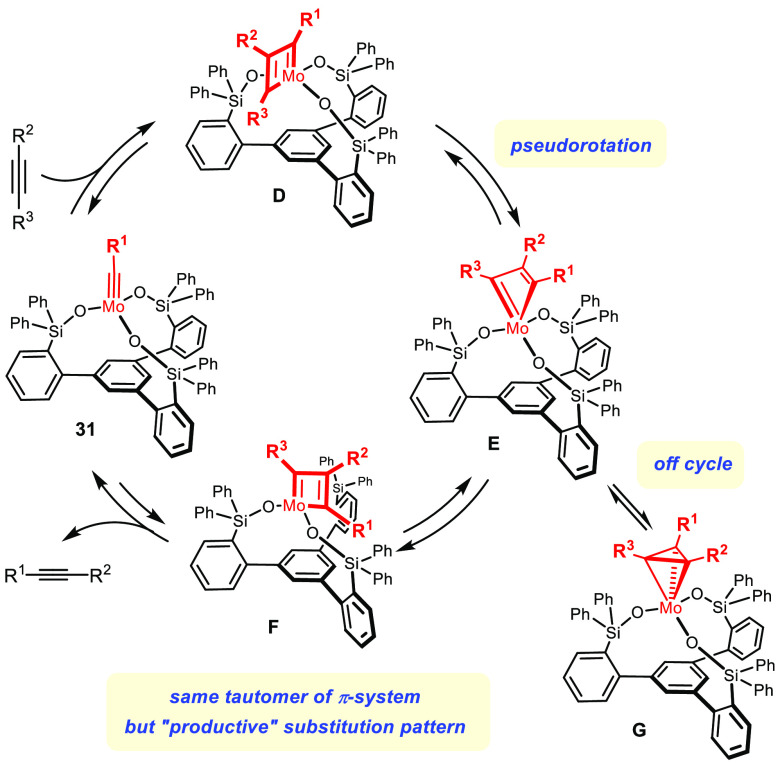
Pseudorotation is Mandatory for Productive Turnover of Canopy Catalysts

Pseudorotation about the adjacent Mo–O bond allows this handicap to be circumvented (Scheme 9):^77,95^ it results in *exchange of the R*^*1*^*and R*^*3*^*substituents on the metallacyclic ring while maintaining the original tautomeric form;* in so doing, an unprecedented gateway to product formation is opened. The actual pseudorotation passes through a “bent” metallacyclic intermediate **E** that can also connect to a metallatetrahedrane **G**.^69,77^ In line with early reasoning, however, **G** was shown to be an off-cycle intermediate, which could even be isolated in certain settings.^94^

A remarkable analogy to observations previously made in the context of alkene metathesis deserves mentioning. In case of Schrock-type molybdenum and tungsten alkylidene complexes, the catalytic cycle passes through a trigonal-bipyramidal metallacyclobutane, which can convert by turnstile rotation into a square-pyramidal isomer with a bent metallacyclic ring;^98^ again, the latter is off the catalytic cycle and presents a potential doorway for catalyst deactivation. The mechanistic similarity to the way the canopy catalysis operates (Scheme 9) is further illustrated by the fact that the catalytically competent metallacycles in ether case are planar and allow the C_α_-atoms to retain noticeable alkylidene (alkylidyne in **D**/**F**) character.^99−101^

## Underappreciated Tools: ^95^Mo and ^183^W NMR

**Figure 7:**
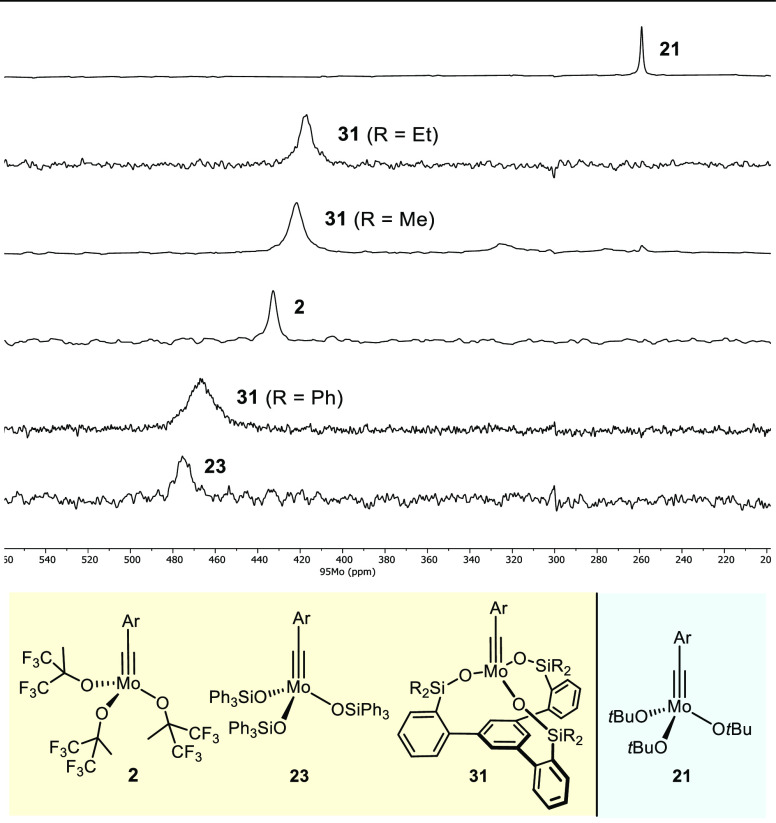
^95^Mo NMR spectra ([D_8_]-toluene, 60 °C) of different molybdenum alkylidyne complexes; Ar = 2,6-Me_2_C_6_H_3_.

The ^13^C NMR data of alkylidynes are informative, especially when the recorded isotropic shift is deconvoluted into the individual components of the shift tensor by computational means.^100^ Such an analysis allows the *energy differences* between filled and empty orbitals (including the HOMO/LUMO gap) to be assessed. Complementary information can be gained by inspecting the other end of the alkylidyne, that is the molybdenum center. Despite a poor gyromagnetic ratio and low abundance, the spin 5/2 nucleus ^95^Mo lends itself for this very purpose;^102,103^ good spectra were obtained in short acquisition times at slightly elevated temperatures to slow down quadrupolar relaxation (Figure 7).

The resonances of the catalytically active complexes **2**, **23**, and **31** are distinct from that of their inactive cousin **21**.^75,93^ If one takes the ^95^Mo chemical shift as a proxy for the Lewis acidity, the comparison shown in Figure 7 is intuitive: qualitatively, it suggests that molybdenum alkylidynes with fluorinated alkoxide or silanolate ligands are notably more Lewis acidic than those comprising ordinary alkoxides, which tallies well with the conclusions drawn from UV/vis and DFT data.^69^ Importantly, ^95^Mo NMR is able to pick up remote and hence subtle effects such as changes in the periphery: thus, δ_Mo_ of complexes **31** with R = Me and R = Ph are no less than 45 ppm apart, whereas their ^13^C NMR signals show little difference (Δδ_C_ = 7.5 ppm). More quantitative interpretations of the ^95^Mo shifts, however, mandate computational assistance. This is particularly true since the electrophilic character of the metal center is strongly geometry-dependent, most notably on the Mo–O–Si bond angles which modulate the donor strengths of the silanolate ligands (see above). For the floppiness of silanolates, the ^95^Mo NMR shifts in solution necessarily average over many conformations. It will therefore be of interest to record complementary solid state ^95^Mo NMR data where the dynamic is largely frozen out and a clearer picture of the inherent nature of the different catalysts might emerge; the experimental challenge, however, is considerable.

## Unequal Twins

**Figure 8:**
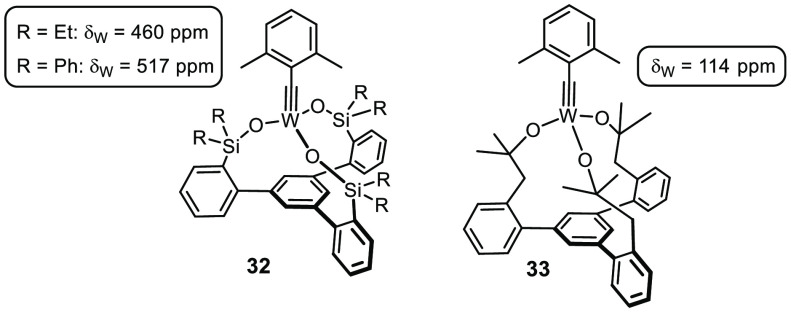
Tungsten alkylidynes with tripodal silanolate or alkoxide ligand frameworks exhibit strikingly different activities.

Whereas (tripodal) silanolates synergize remarkably well with molybdenum alkylidynes, they proved largely inadequate in the tungsten series. In essence, these poor π-donors render the W(+6) center in complexes such as **32** too Lewis acidic (Figure 8). As a consequence, the derived metallacycles are overstabilized and turnover comes to a halt (the same is true for highly fluorinated alkoxide ligands).^69,77,104^ Moreover, competing polymerization of the alkyne substrate is a serious issue.^104^ Once again, it proved highly informative to interrogate the operative transition metal entity directly, in this case via ^183^W NMR, which proved sensitive even to small changes in the chemical environment; the strongly deshielded signals of **32** and congeners are indicative of an overly electrophilic alkylidyne.^104^ This conclusion was confirmed by a computational analysis of the ^13^C shift tensors.

Therefore, the ligand design was revisited and the silicon linkers were replaced altogether.^104^ Although one might think of complex **33** as a tethered variant of the classical Schrock catalyst **1**, the constrained ligand geometry pays valuable dividends. Thus, **33** shows a notably better functional group tolerance than **1**, although it does not rival the best molybdenum canopy catalysts available to date.^97,104^

## Alternative Catalyst Designs

**Scheme 10:**
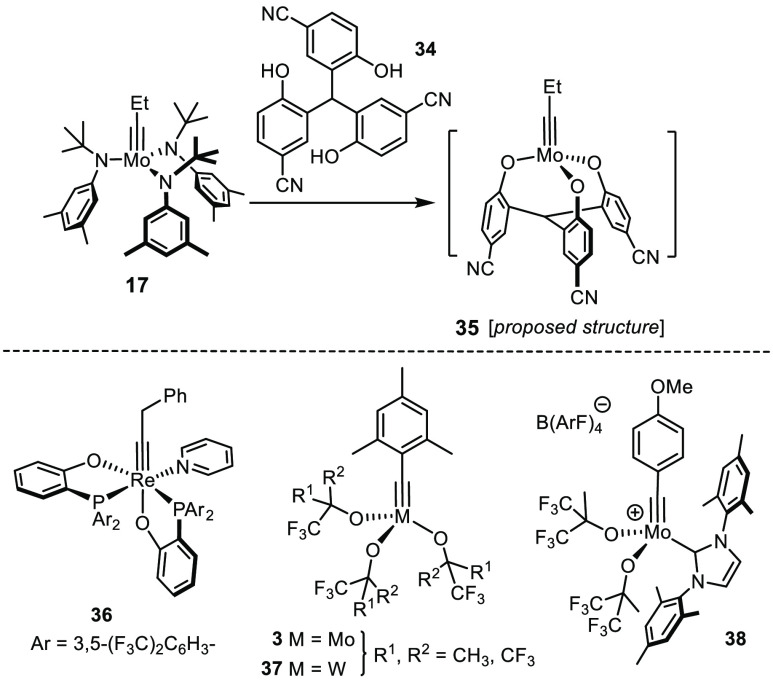
Lexicon of Alternative Catalysts

An alternative design pursued by the Zhang group is also heading toward more tolerant catalysts with chelate ligand frameworks. It relies on phenolate units held together by different types of tethers;^105−107^ the most advanced manifestation features a simple CH group as the central linker (Scheme 10).^108^ The actual catalyst is generated in situ upon reaction of **34** with complex **17**. Although a firm proof of formation of the presumed monomeric species **35** is missing, the reactivity of this two-component system is high enough to allow certain applications to be performed under quasi “open air” conditions; however, CCl_4_ is required as solvent or cosolvent for optimal results.^108^ A number of challenging functional groups were found to be compatible, including pyridine, thiophene, aromatic aldehydes, nitriles, nitro groups, and an arylpinacolboronate. That a phenol-based ligand set is adequate for phenol-bearing substrates is perhaps unsurprising yet worth mentioning.

A recent disclosure by Jia and co-workers deserves special emphasis for two reasons:^109^ first, the d^2^ Re(+5) complex **36** is an extremely rare case of a non-d^0^ alkylidyne showing catalytic activity.^110^ Moreover, **36** is air-stable and tolerates various groups that had been problematic in the past: specifically, it is the only catalyst known to date that works even in the presence of an unprotected carboxylic acid. Other protic groups including primary alcohols, phenols, or aniline are equally compatible, as are certain donor sites.^109^ Although the reactions catalyzed by **36** are rather slow and require high temperatures (≥100 °C in toluene), further scrutiny of this lead compound is warranted.

A myriad of other molybdenum and tungsten alkylidyne complexes was published during the last decades (Scheme 10). The revival of catalysts such as **3** or **37** carrying partly fluorinated or perfluorinated alkoxide ligands has already been mentioned. A “volcano-type” correlation between the degree of fluorination and catalytic activity was established, which has to do with the fact that, from a certain degree of peripheral fluorination onward, the central atoms are too Lewis acidic and the pertinent intermediates on the catalytic cycle overstabilized.^8,111−113^ Yet other catalyst families are distinguished by heteroleptic ligand spheres, comprising, for example, imidazolin-2-iminato or N-heterocyclic carbenes. Some members of this series such as **38** show impressive turnover numbers and rates;^114−119^ their relevance for advanced synthesis, however, is currently difficult to assess as the set of test substrates is (too) narrow and does not contain any of the truly challenging functional groups.

**Scheme 11:**
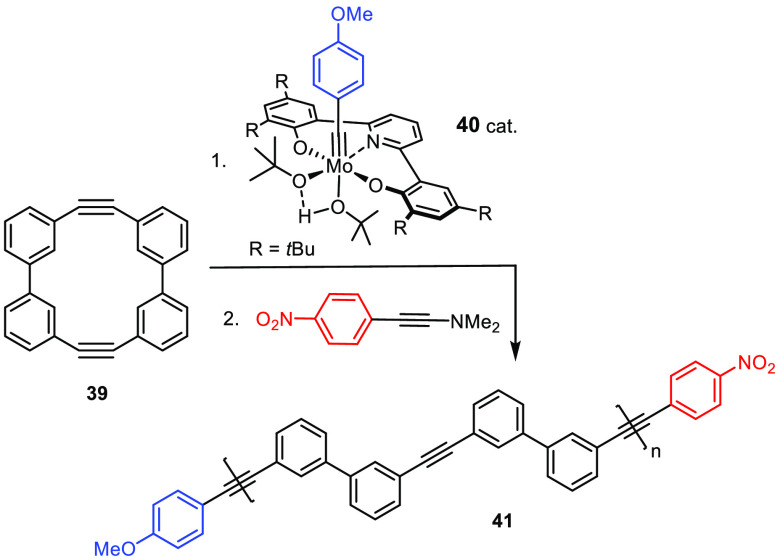
Example of a “Living” ROAMP Reaction

Complex **40** denotes the other extreme (Scheme 11):^120^ its reactivity is tempered to the extent that only a highly strained cycloalkyne such as **39** gets activated, whereas the alkyne units in the resulting polymer **41** remain untouched; for this striking selectivity, chain transfer is precluded. The promise of (living) ring-opening alkyne metathesis polymerization (ROAMP) in general for the formation of precision polymers was recognized only recently;^120−125^ it is expected to provide many opportunities for material science in the future.^5,126^

## Strategy Level Applications

This Perspective does not intend to provide a comprehensive coverage of the applications of alkyne metathesis in organic synthesis and material science. Rather, the following examples are solely meant to illustrate aspects of strategic relevance and encourage further studies in this field.

## Shape-Persistent Objects

Alkyne metathesis is an inherently reversible process: provided the chosen catalyst is sufficiently active and long-lived, the reaction is under thermodynamic control. For this reason, it qualifies for applications to dynamic covalent chemistry (DCC) which mandates that a system is able to correct initial “mistakes” by scrambling of the mixture until the most stable product (distribution) has been reached. In so doing, DCC provides access to molecular objects beyond reach of more conventional approaches.^127^

**Scheme 12:**
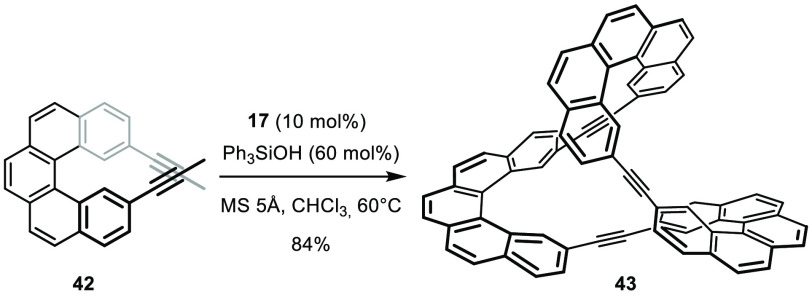
One-Step Formation of a Molecular Möbius Strip

Molybdenum alkylidynes, most notably those supported by silanolate ligands, meet the stringent criteria of activity and stability. The breathtakingly simple and efficient synthesis of **43**, a shape-persistent molecular object of Möbius topology, provides a captivating illustration (Scheme 12).^128^ All it took was to react diyne **42** at 60 °C with a catalyst generated in situ from **17** upon exchange of the original amide ligands for triphenylsilanolates. Together with other similarly intricate applications in the literature,^129−132^ this example suggests that alkyne metathesis has a bright future in the context of material science in general and DCC in particular.^5,126,127^

## π-Bond Selectivity

It is well-known that the standard catalysts for olefin metathesis react with double bonds and triple bonds with similar ease; this indiscriminative behavior is at the very heart of enyne metathesis.^25,133^ In striking contrast, alkyne metathesis catalysts are rigorously selective in that they leave double bonds of all sorts untouched.^134,135^

**Scheme 13:**
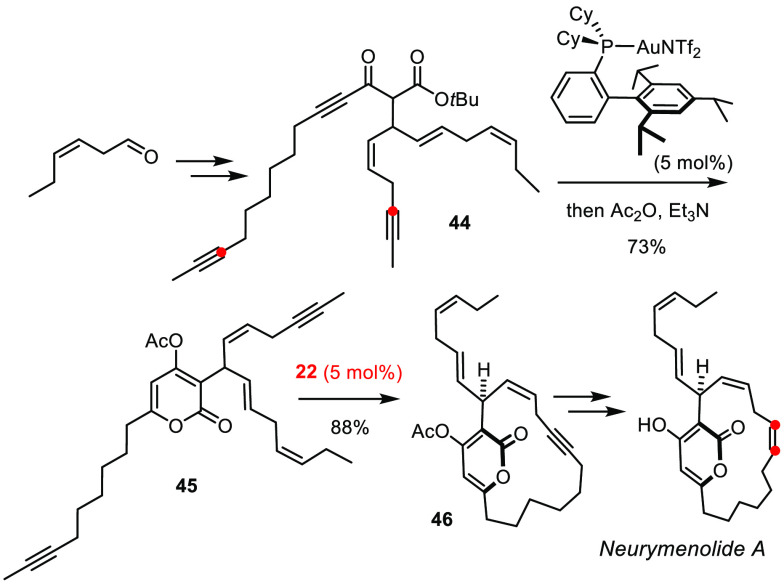
Total Synthesis of Neurymenolide

Two examples must suffice to illustrate how this orthogonality can be leveraged in different chemical context. Neurymenolide A is a structurally unique antibacterial agent and mitotic spindle poison comprising four skipped and hence highly isomerization-prone alkenes. Any attempt to forge the macrocyclic scaffold with the aid of a (*Z*-selective) alkene metathesis catalyst copes with indiscriminate activation of all ((*Z*)-configured) double bonds. RCAM in combination with semireduction allowed the problem to be avoided, and this exceptionally sensitive product to be reached with excellent yield and selectivity (Scheme 13).^136^ Suffice it to say that the power and mildness of gold catalysis for the formation of the 2-pyrone ring was equally critical for success.^137^

**Figure 9:**
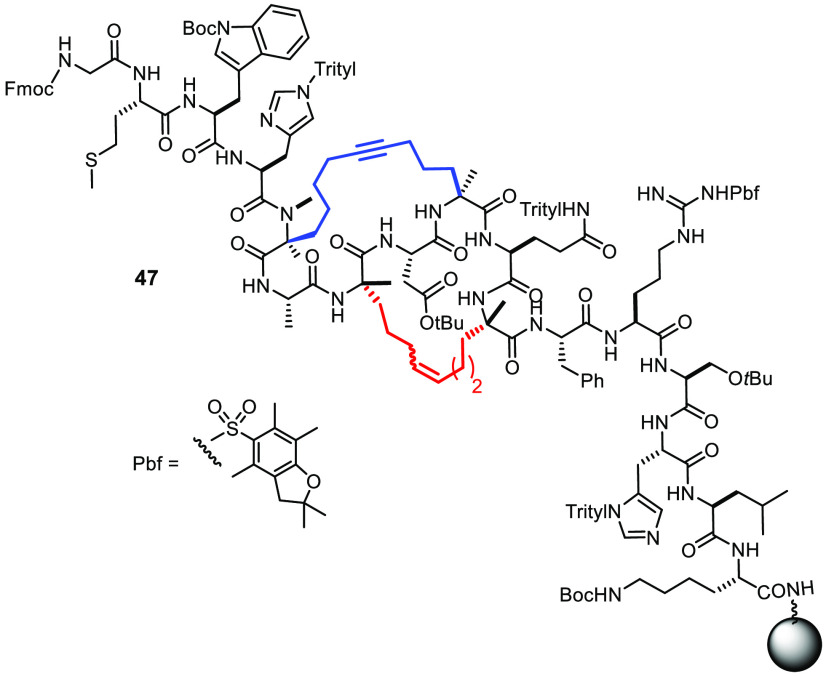
Stapled peptide formed by concomitant yet orthogonal metathetic catenation.

The formation of GTPase targeting stapled peptides by solid-phase synthesis is similarly instructive (Figure 9).^138,139^ Thus, compound **47** comprising an “edge-on” macrobicyclic backbone was obtained in a one-pot operation on reaction of the corresponding diene/diyne substrate with a mixture of first-generation Grubbs catalyst and complex **23**.^138^ This example corroborates the notion that the functional group tolerance of the molybdenum alkylidyne and a classical ruthenium carbene are comparable.

## Trisubstituted Alkenes and Late-Stage Diversity

As described in the Introduction, the formation of stereodefined olefins by alkyne metathesis/semireduction had initially motivated us to engage in this field. In fact, this strategy allowed us to conquer many structurally complex 1,2-disubstituted *Z*- and *E*-alkenes;^6,7,44^ the neurymenolide case alluded to above is representative.

**Scheme 14:**
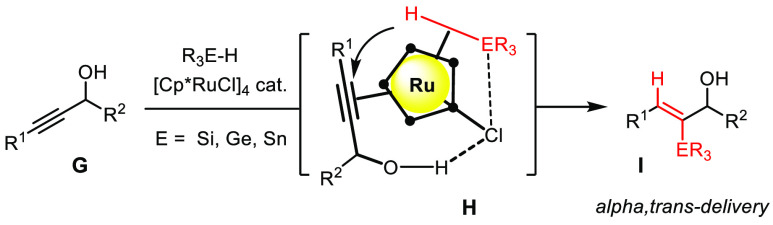
Hydroxy-Directed *trans*-Hydrometalation

It is important to recognize, however, that alkyne metathesis reaches far beyond this initial goal in that it also provides an excellent gateway to trisubstituted alkenes. Much of this progress relates to the fact that propargyl alcohols are compliant and lend themselves to hydroxy-directed *trans*-hydrometalation reactions catalyzed by [Cp*RuCl]_4_ (**G** → **H**, Scheme 14).^140−142^ These stereochemically unorthodox transformations afford products of type **I**, which, in turn, can be elaborated into numerous structural motifs by taking advantage of the rich chemistry of the C_sp2_-ER_3_ (E = Si, Ge, Sn) bond.

**Scheme 15:**
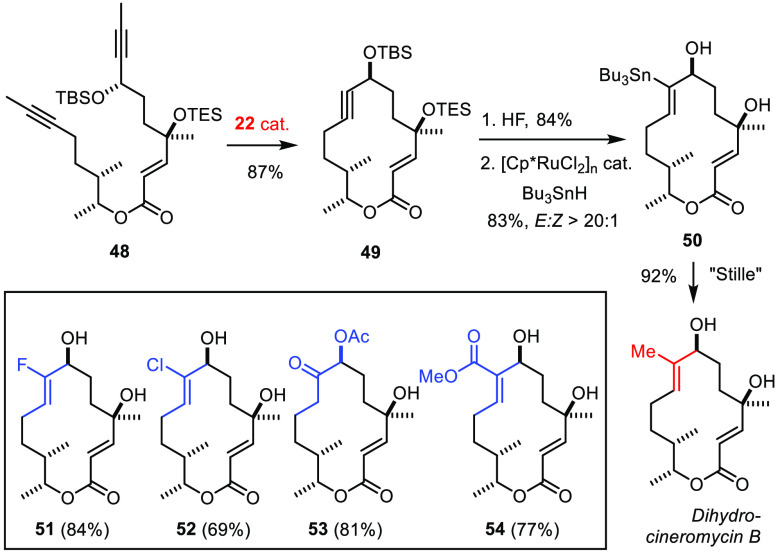
(Diverted) Total Synthesis of a Macrolide Antibiotic

**Figure 10:**
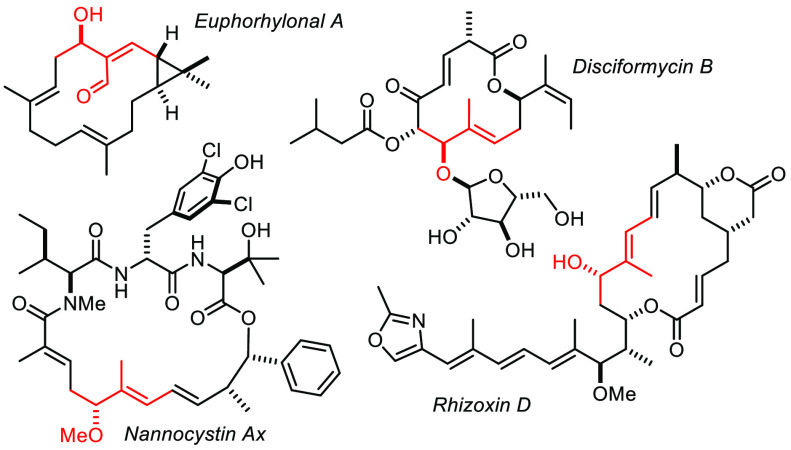
Further applications of RCAM/*trans*-hydrometalation to the synthesis of trisubstituted alkenes.

Ring closure of diyne **48** proceeded well with the molybdenum alkylidyne **22** (Scheme 15).^143^ The resulting propargylic cycloalkyne **49** was transformed into stannane **50**, which was then cross coupled with methyl iodide.^144^ This approach to the antibiotic 5,6-dihydrocineromycin B compares favorably to a previous synthesis in which the macrocycle had been closed at the trisubstituted olefinic site by RCM: 25 mol % of the second-generation Grubbs catalyst was necessary to obtain the target in 40% yield.^145^ As the 2-methyl-but-2-en-1-ol motif is commonplace in natural products, this new and stereoselective approach to trisubstituted alkenes is of more general relevance and has already served other total synthesis endeavors in the polyketide, diterpene, and depsipeptide series (Figure 10).^57,146−148^

At the same time, a metalated intermediate such as **50** provides ample opportunity for late-stage diversification. The small “library” of non-natural analogues **51**–**54** of the dihydrocineromycin estate illustrates this aspect (Scheme 15).^143,149,150^

## A Transannular Rendition

The success of transannular reactions critically hinges on the availability of stereochemically defined macrocyclic precursors. While this “macrocyclic challenge” had been a serious impediment in the past, RCAM in combination with appropriate downstream chemistry is able to revitalize the field.^151^

**Scheme 16:**
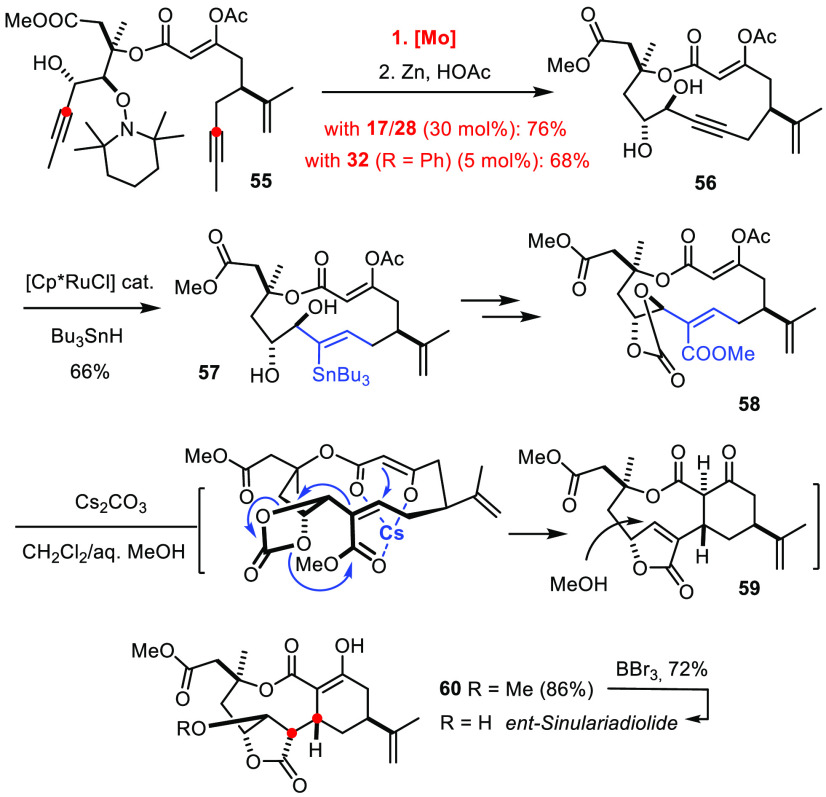
Key Steps of a Total Synthesis of the Nor-Cembranoid Sinulariadiolide

A recent total synthesis of the marine nor-cembranoid sinulariadiolide may illustrate this point (Scheme 16).^152^ Specifically, the intricate tricyclic skeleton comprising an 11-membered nexus was forged by a strain-driven transannular Michael addition reaction. The required precursor **58** comprising a trisubstituted enoate subunit was made in configurationally defined format via RCAM followed by *trans*-hydrostannation/methoxycarbonylation. Ring closure was initially performed with the two-component catalyst system **17**/**28** but later found to be equally efficient with the canopy catalyst **32** developed in parallel in our laboratory.^75^ The compatibility with an unprotected secondary −OH group, a hydroxylamine, an elimination-prone *tert*-aldol substructure, and two different olefinic sites attests to the mildness and chemofidelity of the method. The subsequent ruthenium-catalyzed *trans*-hydrostannation furnished product **57**,^141,142,153^ which was elaborated into the stereodefined Michael acceptor by palladium catalyzed methoxycarbonylation.^154^ The derived cyclic carbonate **58**, on treatment with Cs_2_CO_3_ in MeOH, succumbed to a cascade commencing with cleavage of the enol acetate; this step, in turn, triggered the crucial transannular Michael addition, followed by decarboxylative cleavage of the carbonate, in situ formation of a strained butenolide, and final oxa-Michael addition of external MeOH; as the back-side of the acceptor **59** is shielded by the macrocyclic skeleton, even this intermolecular step proceeded with impeccable selectivity.^152^

Details apart, this example shows that RCAM can help leverage the still underutilized power of transannular reactivity in that it provides a reliable entry into highly decorated and configurationally well-defined macrocyclic substrates. At the same time, it illustrates that alkyne metathesis can make structural patterns available, the descent of which from a triple bond may not be immediately obvious.

## Carbonyl Equivalents

This notion is also exemplified by other similarly intricate case studies. The C atoms of an alkyne have the same formal oxidation state as a carbonyl group, and π-acid catalysis is uniquely capable of harnessing this synthetic equivalence;^155^ once again, transannular settings can help secure the appropriate regioselectivity.^156^

**Scheme 17:**
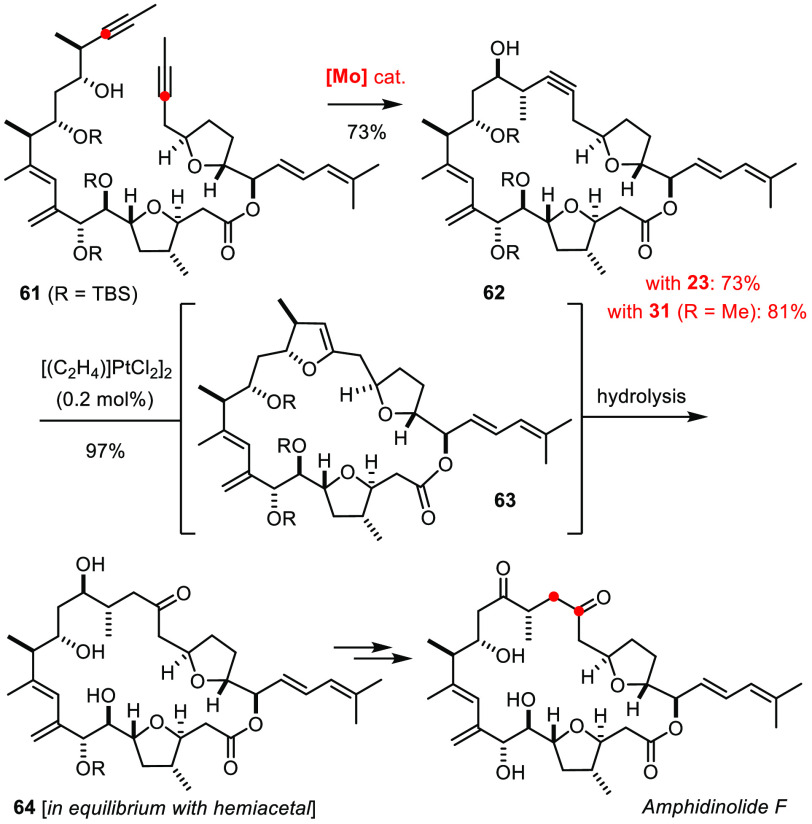
Final Act En Route to Amphidinolide F

The synthesis of spirastrellolide F by RCAM followed by transannular spiroketalization referred to above illustrates this aspect (Scheme 6).^89^ No less challenging is the case shown in Scheme 17:^157,158^ RCAM of the polysubstituted diyne **61** gave product **62**; the reaction was initially performed with **23** as the catalyst and later repeated with the canopy variant **31**.^75^ Activation of **62** with catalytic Pt(+2) entailed a transannular hydroalkoxylation with formation of the labile enol ether **63**, which was hydrolyzed upon workup to reveal the peculiar “umpoled” 1,4-oxygenation pattern of amphidinolide F.^157,158^

**Scheme 18:**
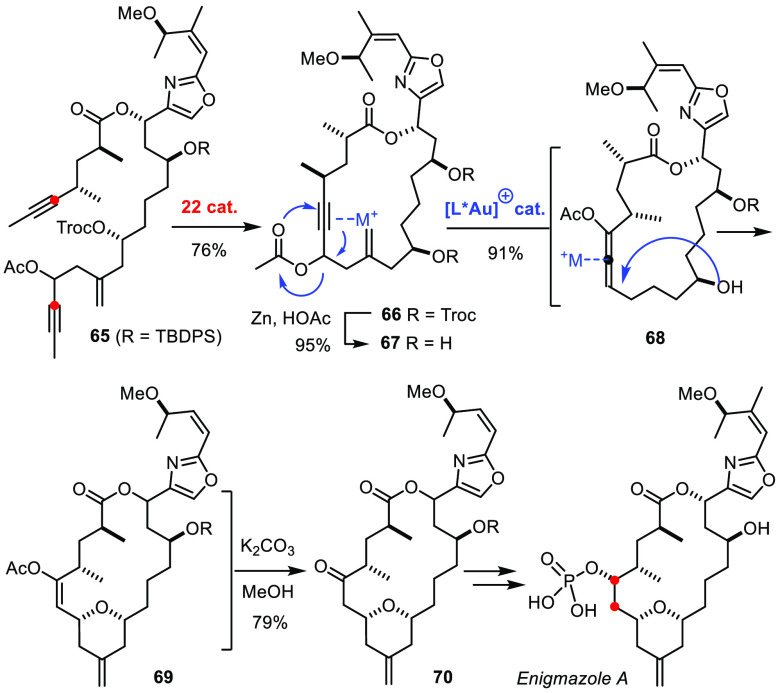
Total Synthesis of Enigmazole A by RCAM in Concert with a Gold-Catalyzed Reaction Cascade

An RCAM-based approach to enigmazole plays with the equivalence of a propargyl acetate and an enone (Scheme 18).^159^ Slow release of the active catalyst **23** from the ate-complex **22** gave the best yield of cycloalkyne **66**, probably because the propargylic acetate in this particular cyclization precursor is exceptionally elimination-prone in the presence of a Lewis acid. Activation of the derived compound **67** with a chiral gold catalyst caused a 3,3-sigmatropic rearrangement with formation of allenyl acetate **68**, which, once formed, gets activated by the very same catalyst and succumbs to transannular hydroalkoxylation. Hydrolysis of the resulting enol ester **69** unveils the Michael addition product **70** as immediate precursor of the targeted natural product.^159^

**Scheme 19:**
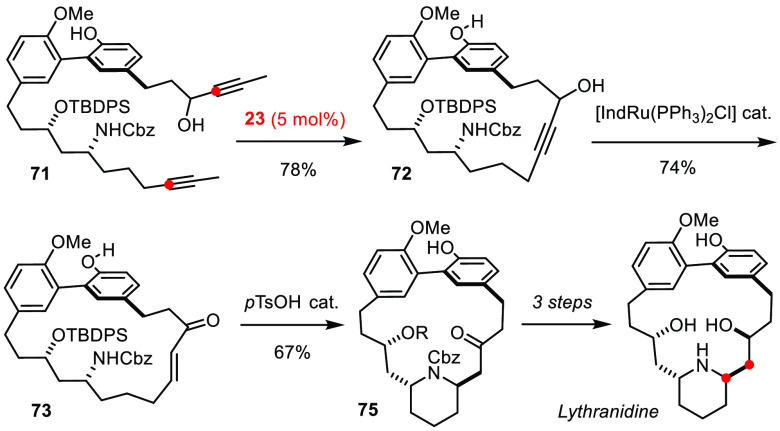
An RCAM/Redox-Isomerization Sequence

Brief reference is also made in this context to lythranidine (Scheme 19).^160^ Once again, it was the tolerance of the molybdenum alkylidyne **23** toward unprotected alcohol and phenol groups that proved enabling. Redox isomerization^161^ of product **72** furnished enone **73** in readiness for a transannular aza-Michael addition. The ability to encode a 1,3-aminoalcohol motif in form of an alkyne may not be immediately apparent. These examples showcase that the cornucopia of alkyne metathesis is filled with a multitude of structural patterns of preparative relevance.

## Heterocyclic Targets

**Scheme 20:**
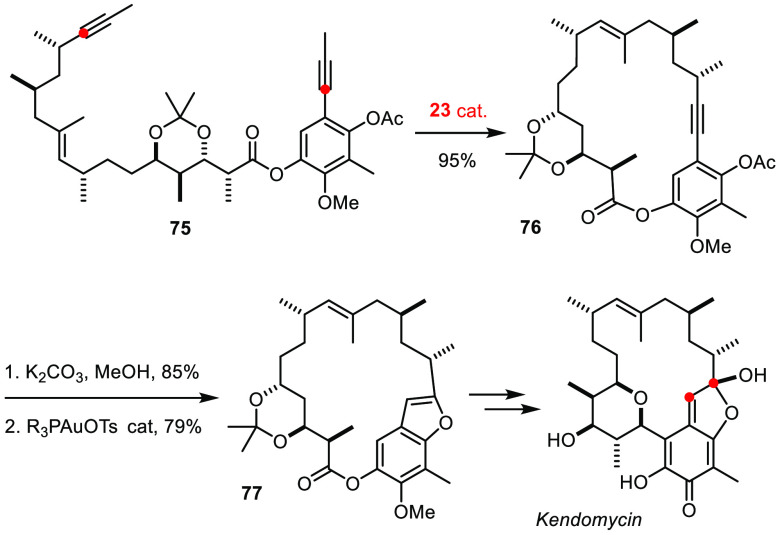
Assembly of Kendomycin at an Unconventional Site

The formation of heterocyclic motifs from alkyne precursors has to be seen in a similar vein. An unconventional approach to the bacterial metabolite kendomycin represents an elaborate example (Scheme 20).^162,163^ RCAM of diyne **75** with formation of **76** set the stage for a subsequent π-acid catalyzed annelation of the benzofuran nucleus (**77**),^164^ which was later oxidized to unveil the quinone-methide/lactol chromophore of this prominent target.

## Terminal Alkyne Metathesis

All examples discussed so far relied on the use of *internal* alkynes; methyl caps are by far most common. An expansion of the substrate pool in general is highly desirable; *terminal* alkynes in particular are potentially lucrative starting points.

The Schrock group had already uncovered why their use is challenging:^165^ the critical step follows the first [2 + 2] cycloaddition in that the resulting complex is prone to transannular C–H insertion with formation of a deprotio-metallacyclobutadiene; this process destroys catalyst and substrate alike. It was much later that Tamm and co-workers noticed that Schrock-type molybdenum alkylidynes **3** endowed with poorly basic hexafluoro-*tert*-butoxide ligands allow this destructive step to be avoided and certain terminal aliphatic alkynes to be metathesized;^166,167^ the chosen test set, however, was small.

Shortly thereafter, complex **23** was found to be equally suited.^65,168^ Upon more comprehensive screening, however, we noticed that this transformation is highly substrate dependent for reasons that are not entirely clear; a late-stage implementation into target-oriented synthesis is therefore still deemed (overly) risky.

**Scheme 21:**
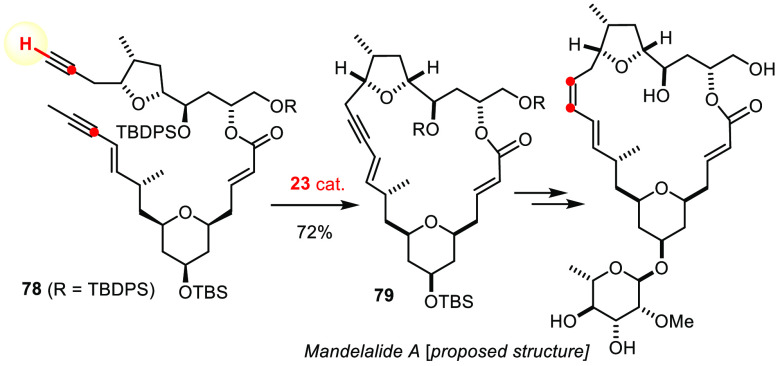
Manifestation of the Reliable Terminal/Internal Alkyne Setting

**Figure 11:**
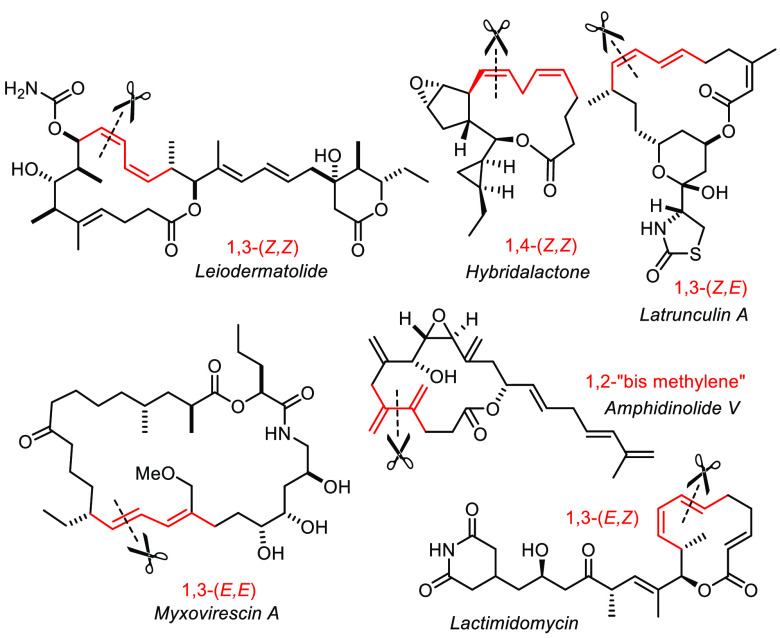
RCAM provides selective entry into all diene motifs.

Gratifyingly, we could show that substrates comprising *one* terminal alkyne and *one* internal alkyne are much better behaved. In this case, RCAM reactions with the aid of **23** and analogues proved robust and high yielding. Our campaign leading to the structure revision of mandelalide A highlights this aspect (Scheme 21).^169,170^ Moreover, this example reiterates the fact that alkene and alkyne metathesis are orthogonal: the 1,3-enyne primarily formed was transformed into the nonthermodynamic *Z,E*-configured 1,3-diene subunit of this particular product. Suffice it to say that enyne/yne metathesis followed by appropriate semireduction *allows all possible 1,3-diene configurations to be reached in a stereoselective manner*, without any scrambing or ring contraction interfering (Figure 11); sensitive skipped 1,4-dienes are equally accessible.^44,136,171−179^ Likewise, 1,3-dienes comprised of *vic*-methylene branches are within reach when RCAM is combined with cross-enyne metathesis with ethylene, as exemplified by the conquest of amphidinolide V.^171,172^

## New Formats

**Scheme 22:**
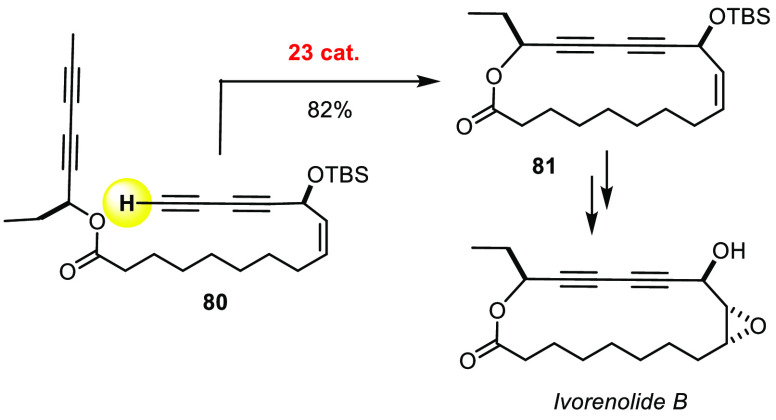
Advanced 1,3-Diyne Metathesis Reaction

Even 1,3-diynes and 1,3,5-triynes can be made by alkyne metathesis, although one might expect that conjugated triple bonds get scambled.^180−182^ This transformation has empowered the total syntheses of the immunomodulatory macrolides ivorenolide A and B (Scheme 22), which further confirm the compliance of substrates comprising one terminal and one internal alkyne.^92,183^

**Scheme 23:**
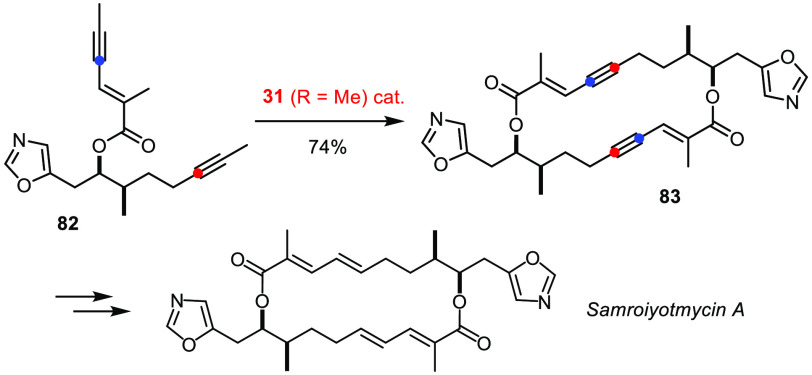
Selective Head-to-Tail Cyclodimerization

Equally useful is controlled head-to-tail cyclodimerization. Recent conquests of disorazole C1^184^ and the antimalarial agent samroiyotmycin A (using the newest canopy catalyst **31**, Scheme 23)^185^ illustrate this reaction format.

## Heteroatom-Containing Triple Bonds

While the power and relevance of catalytic alkyne metathesis is by now largely undisputed, there remains much room for improvement when it comes to reactions of heteroatom-containing triple bonds. Proof-of-concept for nitrile/alkyne metathesis reactions is available;^186^ the major challenge to be met en route to truly efficient settings is the high thermodynamic stability of the resulting metal nitride complexes. The fact, however, that certain such complexes do react with internal alkynes, though fairly slowly and under rather forcing conditions, provides an encouraging outlook.^66,187^

**Scheme 24:**
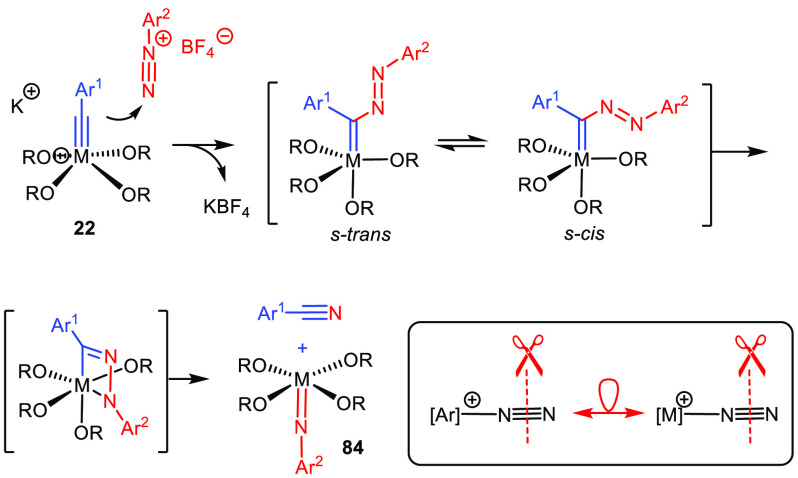
Selective Cleavage of the N≡N Triple Bond of the “World’s Best Leaving Group”

Another unorthodox case is the metathetic activation of N≡N bonds. To the best of our knowledge, all attempts at direct cleavage of molecular nitrogen itself have so far met with failure.^188^ When seen against this backdrop, the exceptional ease with which the triple bond of aryl diazonium salts is activated by ate-complexes **22** (M = Mo, W) is all the more surprising (Scheme 24); the reaction proceeds within minutes even below 0 °C.^189^ For the time being, this reaction is a stoichiometric process, since in situ recycling of the resulting imido complex **84** into an alkylidyne is currently not possible. Although much of the chemistry of aryl diazonium salts is rooted in the exceptional ease with which they lose dinitrogen, **22** exclusively activates the thermodynamically much more stable triple bond. One might hope that this transformation anticipates future cleavage of N_2_, e.g., when bound end-on to an appropriate metal center; if so, it would open a conceptually different foray toward N_2_ activation devoid of any redox steps.

## Conclusions and Outlook

Before the turn of the millennium, alkyne metathesis faced the paradox of being well understood but hardly relevant. This situation has changed since then; the reaction is increasingly recognized as truly enabling and relevant from the strategy point of view; it is clearly more than a subordinate relative of olefin metathesis. At the same time, this development may help correct the common misconception that high-valent early transition metals, other than their “noble” cousins, provide too limited opportunities when polysubstituted, densely functionalized, and/or fragile compounds need to be addressed.

Most applications in the realm of target-oriented synthesis cited above implemented RCAM at a very late stage of a multistep endeavor. The fact that we are willing to subject very precious materials to this methodology illustrates our confidence in the reliability and performance of the catalysts.

The impressive advances of the past decade notwithstanding, a number of issues remain to be tackled. The perhaps most obvious ones concern the current inability to perform reactions in water or other protic media and the still largely missing compatibility with strongly acidic groups. A better availability and even greater ease of handling of the catalysts is also desirable, as are higher turnover numbers when working with (poly)functionalized substrates. Finally, the development of truly catalytic ways of activating heteroatom-containing triple bonds deserves more attention, as such methods would increase the substrate pool to a considerable extent. That the future role of alkyne metathesis will also hinge on the development of ever more effective and selective ways of making and manipulating triple bonds is obvious: π-acid catalysis provides an excellent example for how such concurrent method development can enlarge the scope;^155^ the emergence of catalytic alkyne *trans*- and *gem*-hydrogenation as well as the related *trans*-hydrometalation also needs to be quoted in this context.^140^ Further such advances are desirable, necessary, and arguably feasible.
